# CBX7 negatively regulates migration and invasion in glioma via Wnt/β-catenin pathway inactivation

**DOI:** 10.18632/oncotarget.16587

**Published:** 2017-03-28

**Authors:** Zhongyuan Bao, Xiupeng Xu, Yinlong Liu, Honglu Chao, Chao Lin, Zheng Li, Yongping You, Ning Liu, Jing Ji

**Affiliations:** ^1^ Department of Neurosurgery, The First Affiliated Hospital of Nanjing Medical University, Nanjing, Jiangsu, China

**Keywords:** CBX7, glioma, wnt/β-catenin pathway, DKK1, invasion

## Abstract

CBX7, a member of the Polycomb-group proteins, plays a significant role in normal and cancerous tissues and has been defined as a tumor suppressor in thyroid, breast and pancreatic cancers. However, its function in glioma remains undefined. CBX7 expression is decreased in glioma, especially in higher grade cases, according to data in the CGGA, GSE16001 and TCGA databases. Further experimental evidence has shown that exogenous CBX7 overexpression induced apoptosis and inhibited cell proliferation, colony formation and migration of glioma cells. In this study, we show that the invasive ability of glioma cells was decreased following CBX7 overexpression and CBX7 overexpression was associated with Wnt/β-catenin pathway inhibition, which also decreased downstream expression of ZEB1, a core epithelial-to-mesenchymal transition factor. This reduction in Wnt signaling is controlled by DKK1, a specific Wnt/β-catenin inhibitor. CBX7 enhances DKK1 expression by binding the DKK1 promoter, as shown in Luciferase reporter assays. Our data confirm that CBX7 inhibits EMT and invasion in glioma, which is manifested by influencing the expression of MMP2, MMP9, E-cadherin, N-cadherin and Vimentin in LN229, T98G cells and primary glioma cells (PGC). Furthermore, as a tumor suppressor, CBX7 expression is pivotal to reduce tumor invasion and evaluate prognosis.

## INTRODUCTION

Glioma is the most common form of brain tumor, accounting for approximately 45% of central nervous system (CNS) tumors and 80% of primary CNS malignancies [1]. Despite recent advances, glioma treatments are not curative, no matter the combinations of surgery, chemotherapy and/or radiotherapy used [2, 3]. Thus, it is necessary to further explore the mechanisms of glioma development to find new therapeutic targets for this disease.

Polycomb-group (PcG) proteins are multiprotein complexes that increase or decrease relative gene expression, accelerating a class of epigenetic events and adding another layer of regulation to gene expression [4]. Previous studies have demonstrated that various combinations of PcG protein complexes contribute to neoplastic progression. PcG proteins are classified into two multi-protein complexes: Polycomb repressive complex 1 (PRC1) and Polycomb repressive complex 2 (PRC2). In normal development, interactions between PRC2 and PRC1 block the transcription of targeted developmental regulatory genes [5]. The PcG protein Chromobox homolog 7 (CBX7) is a member of PRC1, and recent studies have suggested that CBX7 balances self-renewal and differentiation in embryonic stem cells (ESCs) and hematopoietic stem cells (HSCs) [6, 7]. In addition, CBX7 activity has been reported to be negatively correlated to malignancy grade in breast [8], thyroid [9], pancreatic cancer [10], prostate cancer [11], bladder cancer [12], lung cancer [13] and colon cancer [14]. The function of CBX7 may occur at a transcriptional level since quantitative RT-PCR analysis showed a reduced CBX7-specific mRNA levels in colorectal cancer samples versus normal counterpart tissue (up to more than 50-fold) and cyclin E was upregulated in CBX7-KO mice in the lung cancer recearch. Previous research has indicated that upregulated expression of CBX7 inhibited the growth of GBM cells and reduced the expression of CDK2 and cyclin A2 (CCNA2). Overexpression of CBX7 significantly recovered the increase in cell proliferation and cell cycle distribution. In consideration of these data, we further investigated the role of CBX7 in glioma on migration and invasion especially.

The Wnt/β-catenin signaling pathway is a crucial mechanism for cellular maintenance and development that includes cell cycle progression, apoptosis, differentiation, migration and proliferation [15]. The Wnt/β-catenin pathway is also stimulated in breast, colon, pancreatic and brain (including glioma) cancer development [16–19]. It is one of the core signaling pathways that influences the balance of cancer stem cell (CSC) self-renewal, which activates and maintains malignant tumors with stem cell characteristics [20]. Dysregulation of the Wnt pathway is correlated with oncogenesis in various tissues including breast, colon, pancreas and CNS. During neoplastic transformation, the Wnt/β-catenin pathway is often aberrantly activated, increasing migration and invasion characteristics through increased β-catenin phosphorylation and/or nuclear localization [21]. Furthermore, the Wnt/β-catenin pathway can induce epithelial-to-mesenchymal transition (EMT) as active Wnt/β-catenin signaling triggers the expression of a set of EMT activators, including Twist1, ZEB1, Snail and Slug [22]. Here, we found that CBX7 is a critical tumor suppressor that silences Wnt/β-catenin signaling; CBX7 overexpression decreases nuclear β-catenin levels and deblocks translocation from the nucleus to the cytoplasm, while minimally affecting total β-catenin levels. This effect is mediated by DKK1, a Wnt/β-catenin inhibitor [23]. CBX7 increases DKK1 transcription via binding the DKK1 promoter, as shown by chromatin immunoprecipitation (ChIP) assays. CBX7 overexpression also resulted in reduced ZEB1 expression, a downstream target of the Wnt/β-catenin pathway and a classical EMT regulator in many types of cancer. This decrease in ZEB1 was associated with decreased *in vivo* and *in vitro* migration and invasion. Thus, this study highlighted a novel process in glioma development and identified a potential therapeutic target for this common CNS malignancy.

## RESULTS

### CBX7 expression is decreased in glioma and correlated with malignancy grade

**Figure 1:**
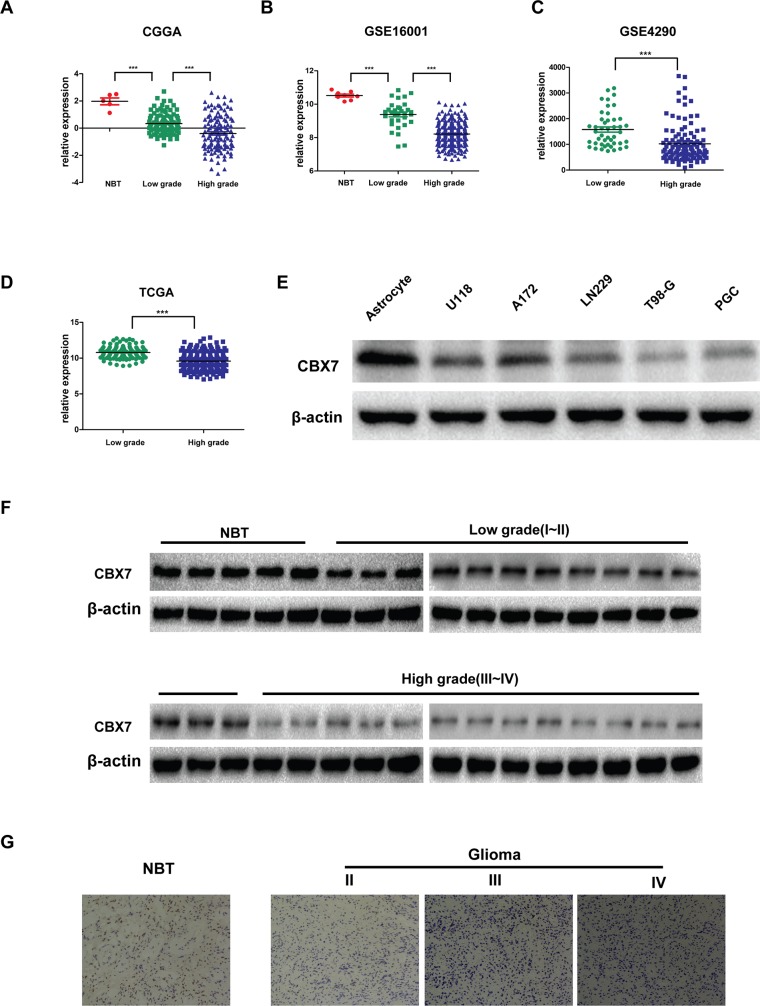
Decreased CBX7 expression confers high-grade glioma **(A-B)** The expression of CBX7 was analyzed in normal brain tissues and glioma tissues of the CGGA (n=225) and GSE16001 (n=284) glioma datasets. **(C-D)** The levels of CBX7 were analyzed in glioma tissues of the GSE4290 (n=181) and TCGA (n=426) glioma database. **(E)** The expression of CBX7 is detected in normal astrocyte and several glioma cell lines concluding U118, A172, T98G, LN229 and PGC by western blot. **(F)** The expression of CBX7 is tested in normal brain tissues and glioma tissues with 4 grades from Jiangsu Province Hospital by Western blot. **(G)** The expression of CBX7 is tested in normal brain tissues and glioma tissues (II-IV grade) from Jiangsu Province Hospital by Immunohistochemistry. * P<0.05; ** P < 0.01 and *** P < 0.001.

Previous studies have reported decreased CBX7 expression in thyroid, breast and pancreatic cancer, and the effects of its loss indicate CBX7 is a tumor suppressor. However, the role of CBX7 in glioma remains unclear. To investigate potential roles for CBX7 in glioma, we first studied CBX7 expression in human glioma databases. As shown in Figure 1A and 1B, database analysis revealed the presence of CBX7 protein in normal brain tissues and glioma tissues, but lower expression in glioma tissues [24]. Moreover, higher grade disease was correlated with lower CBX7 expression (Figure 1A–1D). Five glioma cell lines, including primary glioma cells (PGC) established in March 2016 from tumor cells taken from a patient with a left tempus glioblastoma, and one astrocyte cell line were then examined for CBX7 expression, and the data showed that CBX7 expression was higher in the astrocyte cell line than any of the five glioma cell lines (U118, A172, T98G, LN229 or PGC) (Figure 1E). We then examined CBX7 expression in normal and glioma samples from Jiangsu Province Hospital. Both western blotting and immunohistochemistry assays indicated that CBX7 expression was highest in normal tissues and gradually decreased as glioma malignancy grade increased (Figure 1E and 1F). These results are consistent with database analyses. In conclusion, CBX7 expression is highest in normal tissues and lower expression is seen in glioma, which might contribute to increased malignancy.

### Exogenous CBX7 expression reverses malignant phenotypes of glioma cells

**Figure 2:**
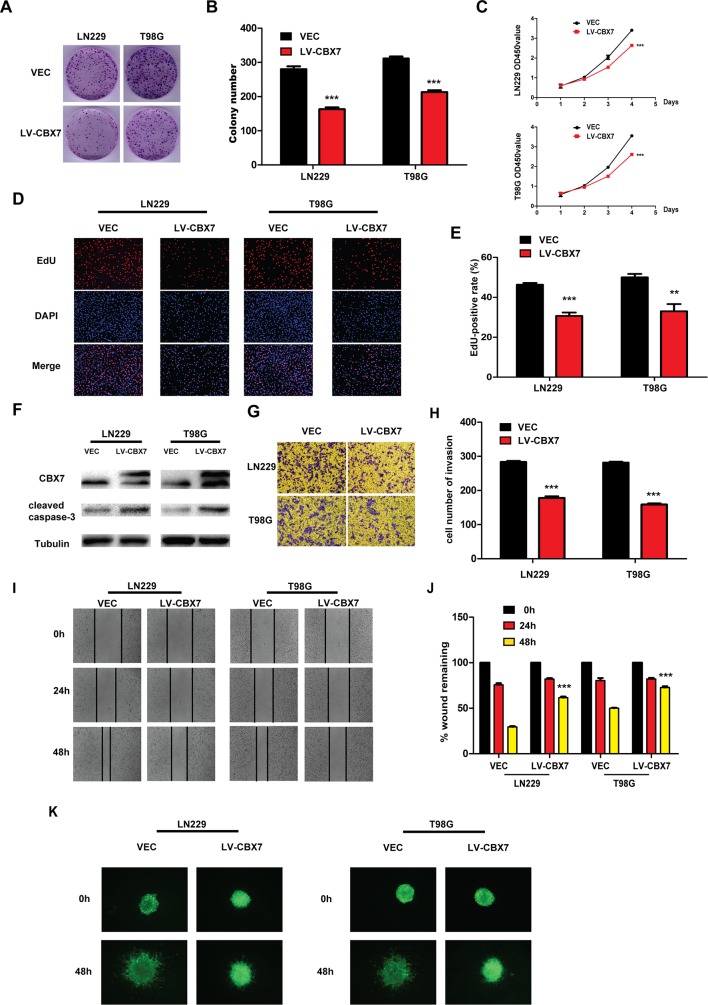
CBX7 depresses the different tumorigenic degree of glioma cells **(A-B)** LN229 and T98G were treated with LV-CBX7 and performed colony formation assay. LN229/VEC, T98G/VEC, LN229/CBX7 and T98G/CBX7 stabled cells were counted and plated at a density of 400 cells per well and allowed for colony formation. Colonies were stained with crystal violet. Mean colony counts are displayed. Transfected cells exhibited a significant reduction of colony formation after 2 weeks. **(C)** CCK-8 assay displays decreased proliferation in LN229 and T98G after overexpressing CBX7. **(D-E)** The EDU showed repressed growth of LN229 and T98G after CBX7 overexpression. **(F)** The western blot indicates more of cleaved caspase-3 in LN229 and T98G after CBX7 overexpression, indicating more apoptosis. **(G-J)** After transfecting LV-CBX7, debilitated ability of migration and invasion was demonstrated by wound-healing assay and transwell assay. **(K)** Three-dimensional spheroid assays was carried out to display invasive ability after overexpressing CBX7. All experiments were performed in triplicate. * P≤0.05; ** P ≤ 0.01 and *** P ≤ 0.001.

Next, three glioma cell lines, LN229, T98G and PGC, were selected to investigate phenotypic changes following CBX7 overexpression. LN229, T98G and PGC cells were transduced with a lentivirus carrying CBX7 to form stable CBX7-overexpressing cell lines. It has been proven that CBX7 controls the growth and self-renewal of normal and tumor-derived prostate cells [25]. Colony formation and CCK-8 assays were performed after transfecting cells. We detected significant inhibitory effects on the viabilities of LN229 and T98G cells compared with the controls (Figure 2A, 2C). The statistical result of colony formation was shown in Figure 2B. Further, when we used EDU cell-image assay to evaluate the effect of CBX7 on proliferation, the results were similar to those acquired using the CCK-8 and colony formation assays. Thus, the EDU-positive rates of LN229 and T98G cells were lower compared with those of the controls (Figure 2D), which was displayed statistically in Figure 2E.

The results show that proliferation is decreased following CBX7 overexpression (Figure 2A–2E). High levels of cleaved caspase-3 in CBX7-overexpressing LN229 and T98G cells indicated that apoptosis was also induced (Figure 2F). LN229-CBX7 and T98G-CBX7 stable cells also showed reduced invasion and migration through transwell and wound-healing assays (Figure 2G and 2I). The statistical data was in Figure 2H and 2J. We also used three-dimensional spheroid assays to analyze the invasive ability of transfected cell lines. As shown in Figure 2K, CBX7-overexpressing cells moved slower than empty vector controls. For further exploration of the cell motion after transfection, PGC was referred to perform wound-healing, transwell and three-dimensional spheroid assays. Low capability of motion was presented in transwell and wound-healing assays (Supplementary Figure 1A and 1C). Results were shown in Supplementary Figure 1B and 1D statistically. In three-dimensional spheroid assays, less PGC-CBX7 moved out from spheroid than PGC-VEC. (Supplementary Figure 1E).

### Decreased invasion after CBX7 overexpression is associated with reduced EMT and Matrix metalloproteinase (MMP) expression

**Figure 3:**
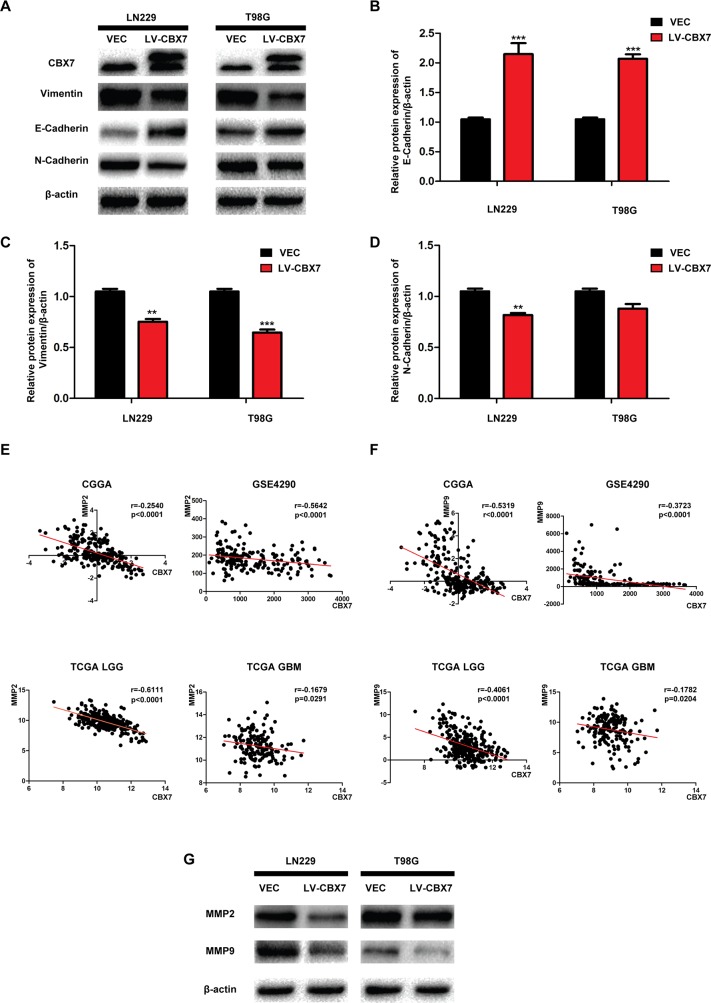
CBX7 overexpression inhibits glioma invasion in protein level **(A-D)** Cells were transfected with CBX7, and the levels of cell EMT relative proteins were detected by western blot analysis. β–actin was used as an endogenous normalizer. **(E-F)** The correlation between CBX7 and MMP2 or MMP9 was analyzed by GSE4290, CGGA and TCGA database. **(G)** Western blot assay was used to show decreased expression in LN229/CBX7 and T98G/CBX7 stabled cells. * P<0.05; ** P < 0.01 and *** P < 0.001.

EMT is a reversible biological process that occurs in epithelial cells during normal development and aberrantly in many cancers [26]. EMT ultimately leads to the acquisition of a mesenchymal phenotype, which is characterized by increased cell motility and resistance to genotoxic agents [27]. Vimentin and N-Cadherin are classic markers of this mesenchymal phenotype and are used to identify cancer tissues undergoing EMT [28]. The epithelial marker E-Cadherin is rarely expressed in gliomas and positively correlates to CBX7 expression, as previously demonstrated by ChIP assays in other cancer types [29, 30]. We first performed western blotting to confirm this relationship in LN229-CBX7, T98G-CBX7 and PGC-CBX7 cells. Three FLAG-tags were fused to the exogenous CBX7, allowing quantitation of the additional CBX7 expression relative to endogenous (Figure 3A and Supplementary Figure 1F). As shown in Figure 3A–3D, E-Cadherin expression was increased in CBX7-overexpressing cells compared with the parental cell lines; in contrast, Vimentin was down-regulated. N-Cadherin expression was significantly decreased in LN229-CBX7 stables but in T98G-CBX7 it was not obviously changed. This difference may be related to differences in these specific cell lines. Similar results, high expression of E-Cadherin and low in Vimentin and N-Cadherin after CBX7 transfection, were verified in PCG. In general, the EMT-like process, because of little epithelial origin in glioma, was decreased following CBX7 expression, as evidenced by the expression of epithelial and mesenchymal markers in these cell lines [31]. MMPs are a family of more than 28 enzymes that were initially identified on the basis of their ability to break most elements of the extracellular matrix (ECM), but have subsequently been found to be upregulated in nearly every tumor type [32]. As ECM breakdown is essential for tumor invasion and metastasis, MMPs have been studied for their role in these later stages of tumor development. More recently, these enzymes have been found to impact cellular signaling pathways that stimulate cell growth during the early stages of tumor development. Vast evidence indicates that tumor-associated MMPs can also promote processes associated with EMT [33]. We chose in investigate MMP2 and MMP9 expression in the CGGA, GSE4290 and TCGA databases to analyze the correlation between CBX7 and MMP expression. This analysis indicated that MMP2 and MMP9 were negatively regulated by CBX7 (Figure 3E and 3F). To experimentally investigate this relationship, relative MMP2 and MMP9 expression was tested in LN229-CBX7, T98G-CBX7 and PGC-CBX7 stable cells by western blot (Figure 3G and Supplementary Figure 1J). The results showed that MMP9 expression was significantly reduced after CBX7 overexpression. However, MMP2 was not down-regulated in T98G-CBX7 stables to the same degree as in LN229-CBX7 stables, indicating that other factors may be influencing MMP2 expression in the T98G cell line. Overall, these data show that CBX7 overexpression reduces the migration and invasion capabilities of glioma cells.

### CBX7 overexpression decreases Wnt/β-catenin signaling, blocking glioma cell invasion

**Figure 4:**
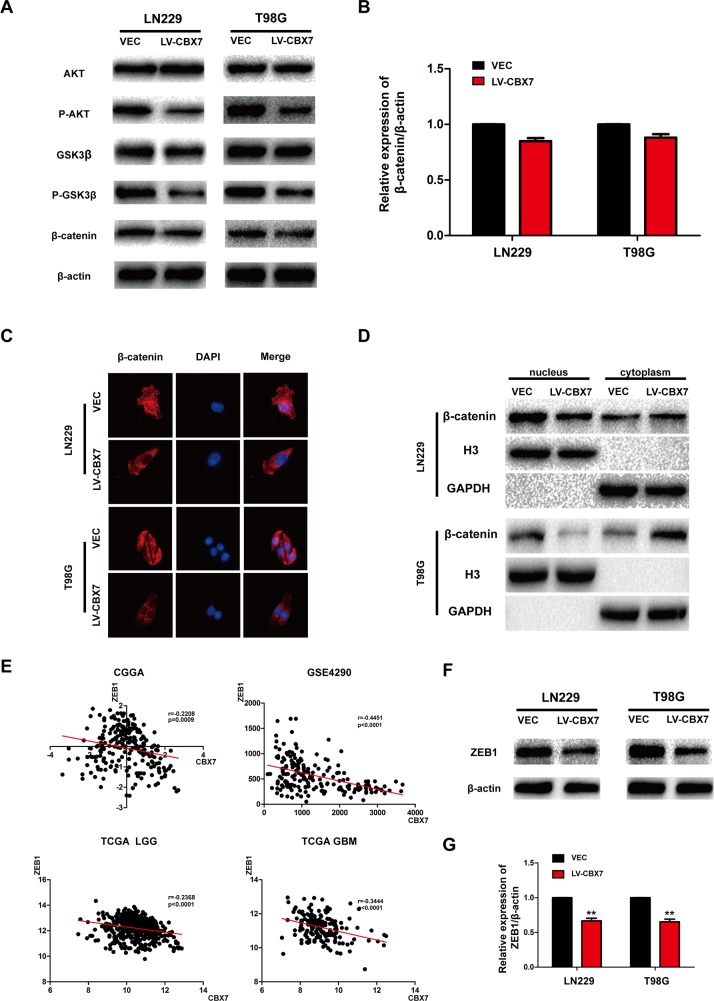
Effect of CBX7 on the activity of the Wnt/β-catenin pathway **(A)** The effect of CBX7 on the Wnt signaling pathway was analyzed by immunoblotting with the indicated antibodies. **(B)** Difference of total β-catenin expression is presented statistically. **(C)** Cells were stained with an anti-β-catenin antibody (red) and DAPI (blue). **(D)** Cells were fractionated, and the nuclear and cytoplasmic extracts were subjected to immunoblotting. GAPDH and H3 were used as controls for the cytoplasmic and nuclear, respectively. The top three in Figure 4D correspond to LN229 and the bottom three correspond to T98G. **(E)** GSE4290, CGGA, TCGA database were used to analyzed the correlation between CBX7 and ZEB1. **(F-G)** ZEB1 expression in LN229/CBX7 and T98G/CBX7 stabled cells were subjected to immunoblotting. β-actin was used as an endogenous normalizer. * P<0.05; ** P < 0.01 and *** P < 0.001.

The Wnt/β-catenin pathway regulates diverse cellular functions including proliferation, adhesion, survival and differentiation, and tumor occurrence is closely related to dysregulated Wnt/β-catenin signaling. The Wnt/β-catenin pathway also plays a critical role in EMT in glioma cells. Wnt signaling is initiated when Wnt ligands bind to transmembrane receptors of the Frizzled family, which stabilize β-catenin by inhibiting the APC complex. As a result, β-catenin accumulates in the nucleus, forming the β-catenin/TCF/LEF transcriptional complex, which activates Wnt target genes and promotes EMT [30]. Therefore, inhibiting Wnt/β-catenin signaling could block EMT and reduce cancer cell invasion. To test the effect of CBX7 overexpression on Wnt/β-catenin signaling, relative protein levels were analyzed by immunoblotting. Total levels of AKT and GSK3-β did not show obvious differences between CBX7-overexpressing and parental cells, but AKT and GSK3β phosphorylation were decreased in LN229-CBX7, T98G-CBX7 and PGC-CBX7 stable cells (Figure 4A and Supplementary Figure 1K). Although total β-catenin levels were not significantly altered (Figure 4B), CBX7 overexpression resulted in reduced nuclear β-catenin levels and increased cytoplasmic levels, as demonstrated by immunoblotting nuclear and cytoplasmic fractions and immunofluorescence staining (Figure 4C, 4D and Supplementary Figure 1L,1M). In most cancer types, β-catenin is sequestered in the cytoplasm by E-cadherin, and the translocation of β-catenin into the nucleus follows E-cadherin downregulation, and thus, is directly correlated with the acquisition of mesenchymal phenotypes [34]. CBX7-overexpressing cells showed decreased nuclear β-catenin, which correlated with reduced EMT phenotypes.

ZEB1, a member of the zinc-finger E-box-binding homeobox (ZEB) protein family, is a core factor responsible for mediating EMT in numerous cancer types, including glioma. Moreover, ZEB1 is a target of the Wnt/β-catenin pathway [35]. Correlations between CBX7 and ZEB1 expression were analyzed in the CGGA, GSE4290 and TCGA databases, and we found a negative correlation between the expression of the two genes (Figure 4E). These results were corroborated in LN229, T98G and PGC cells by immunoblotting (Figure 4F, 4G and Supplementary Figure 1N). What's more, in order to exam variation of other EMT markers, Slug, Snail, Twist and ZO-1 were tested in LN229 and T98G (Supplementary Figure 4A and 4C) and counted (Supplementary Figure 4B and 4D). There was no variation except decreased Slug in LN229, which may exist difference in different cell lines. No variation was detected in PGC, either (Supplementary Figure 1O). In conclusion, CBX7 not only decreased Wnt/β-catenin activity, but also downstream ZEB1 expression, which subsequently blocked EMT and invasion.

### CBX7 enhances DKK1 expression by binding to its promoter

**Figure 5:**
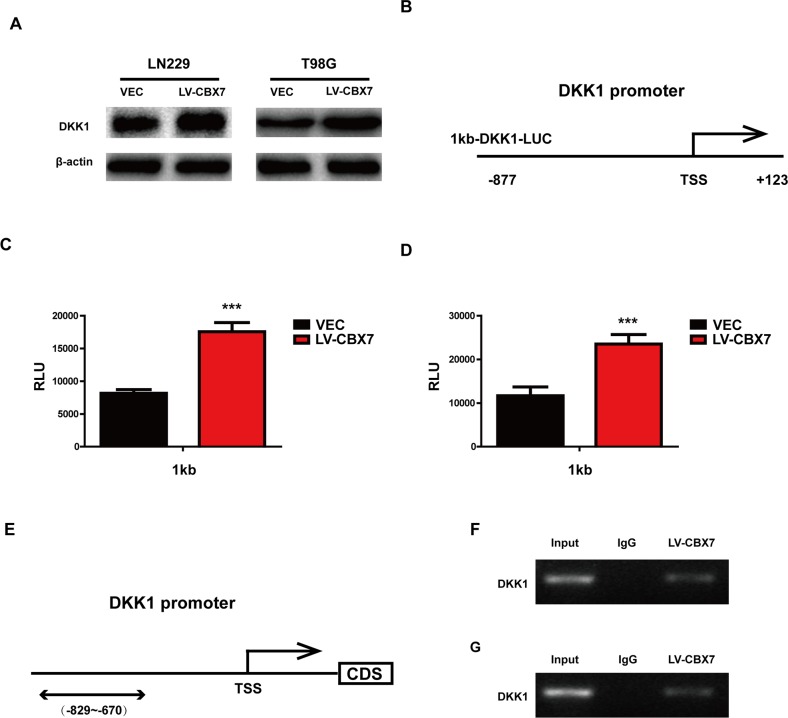
CBX7 enhanced DKK1 expression by binding promoter **(A)** CBX7 impact the expression of DKK1, analyzed by immunoblotting with the indicated antibodies. β-actin was used as control. **(B-D)** A luciferase reporter assay was performed to measure DKK-1 promoter activity in LN229 and T98G. TSS, transcription start site. **(E)** Schematic illustration of the promoter regions of the human DKK-1 gene and the regions containing the primers for ChIP assay (under). **(F)** The results of ChIP analysis show the CBX7 was recruited to the DKK-1 promoter regions in CBX7-overexpressing LN229 and T98G cells. IgG was used as an immunoprecipitation control. * P≤0.05; ** P ≤ 0.01 and *** P ≤ 0.001.

Dickkopf-1 (DKK1), a secreted Wnt antagonist, inhibits Wnt-induced β-catenin stabilization and the β-catenin/TCF-dependent transcription of both artificial and endogenous genes. In LN229, T98G and PGC cells, maintaining CBX7 expression increased DKK1 expression (Figure 5A and Supplementary Figure 2A). Previous studies have found that CBX7 positively regulates DKK1 transcription via enhancing histone acetylation in breast the cancer cell line MCF7 [8]. To examine whether CBX7 up-regulated DKK1 expression at the transcriptional level in glioma, luciferase reporter assays were performed to measure DKK1 promoter activity (Figure 5B-5D and Supplementary Figure 2B). We generated luciferase reporter constructs containing 1 kb of the DKK1 promoter for these assays. The CBX7-overexpressing LN229, T98G and PGC cells showed strongly enhanced 1 kb DKK1 promoter activity, indicating that CBX7 upregulated DKK1 expression at the transcriptional level via the DKK1 promoter. Moreover, CBX7 can directly bind to the specific region of the DKK1 promoter in LN229 and T98G, as determined by ChIP assay (Figure 5E and 5F). A ChIP assay was also performed in PGC and similar results were detected in Supplementary Figure 2C.

### Decreased Wnt/β-catenin signaling following CBX7 overexpression involves DKK1 activity

**Figure 6:**
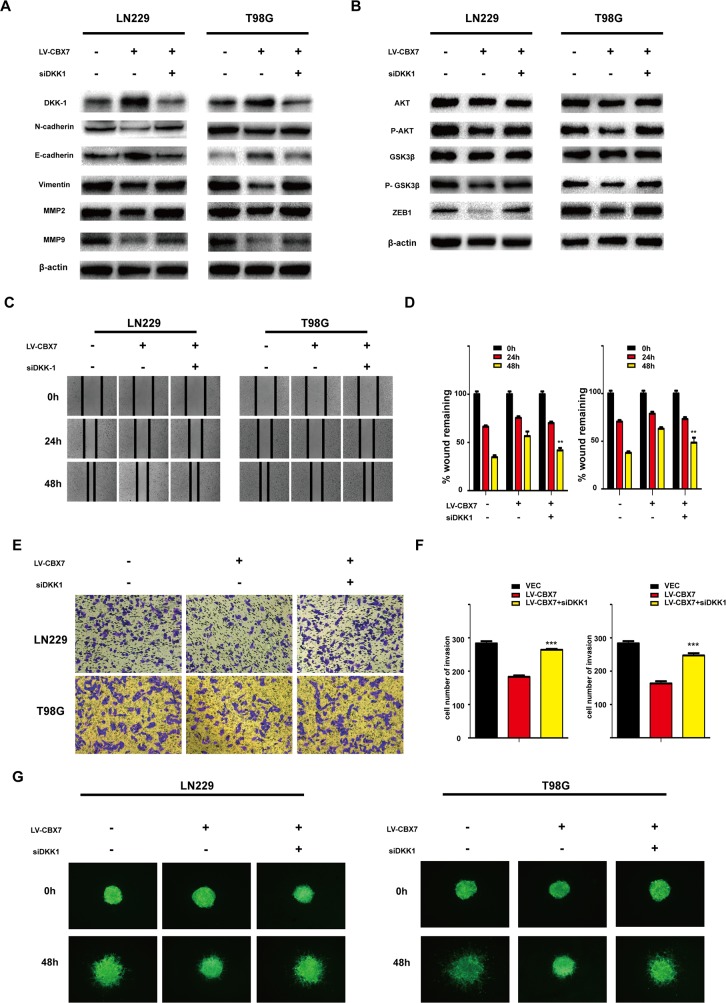
DKK-1 regulates the CBX7-mediated Wnt signaling pathway **(A)** Effect of the inhibition of DKK-1 on CBX7-induced increased activity of the E-Cadherin and oppositively on other invasion related proteins. Cells treated with DKK-1 siRNA were subjected to immunoblotting with appropriate antibodies. **(B)** CBX7-induced reduction of phosphorylated AKT and GSK3-β and ZEB1 were reversed by treatment of siDKK1. β-actin was used as an endogenous normalizer in immunoblotting. **(C-D)** Wound-healing assay, **(E-F)** Transwell assay and **(G)** three-dimensional spheroid assays were performed in LN229 and T98G treated with siDKK1. All experiments were performed in triplicate. * P≤0.05; ** P ≤ 0.01 and *** P ≤ 0.001.

DKK1 is a negative regulator of the Wnt/β-catenin pathway in multiple tumor types and has been classified as a tumor suppressor for this role [36]. We subsequently investigated whether DKK1 is a negative regulator of Wnt signaling in glioma cells overexpressing CBX7. The CBX7-induced reduction of AKT and GSK3-β phosphorylation was reversed by small interfering RNA (siRNA) targeting DKK1, while total AKT and GSK3-β levels were unchanged (Figure 6B and Supplementary Figure 2J). Vimentin and N-Cadherin levels were also enhanced in LN229-CBX7, T98G-CBX7 and PGC-CBX7 stable cells after siDKK1 treatment. In contrast, E-Cadherin expression was reduced, as indicated by western blotting (Figure 6A and Supplementary Figure 2I). Similarly, MMP2, MMP9 and ZEB1 expression were also enhanced, indicating that EMT was activated. The invasion and migration capabilities of LN229 and T98G cells overexpressing CBX7 were also recovered after siDKK1 treatment, as analyzed through wound-healing, transwell and three-dimensional spheroid assays (Figure 6C, 6E, 6G and Supplementary Figure 2D, 2E, 2H) and counted (Figure 6D, 6F and Supplementary Figure 2F, 2G). Together, these data demonstrate that siRNA-mediated DKK1 knockdown antagonizes CBX7 activities; thus CBX7 function is mediated by DKK1.

### CBX7 function is proved in orthotopic and subcutaneous nude mice model

**Figure 7:**
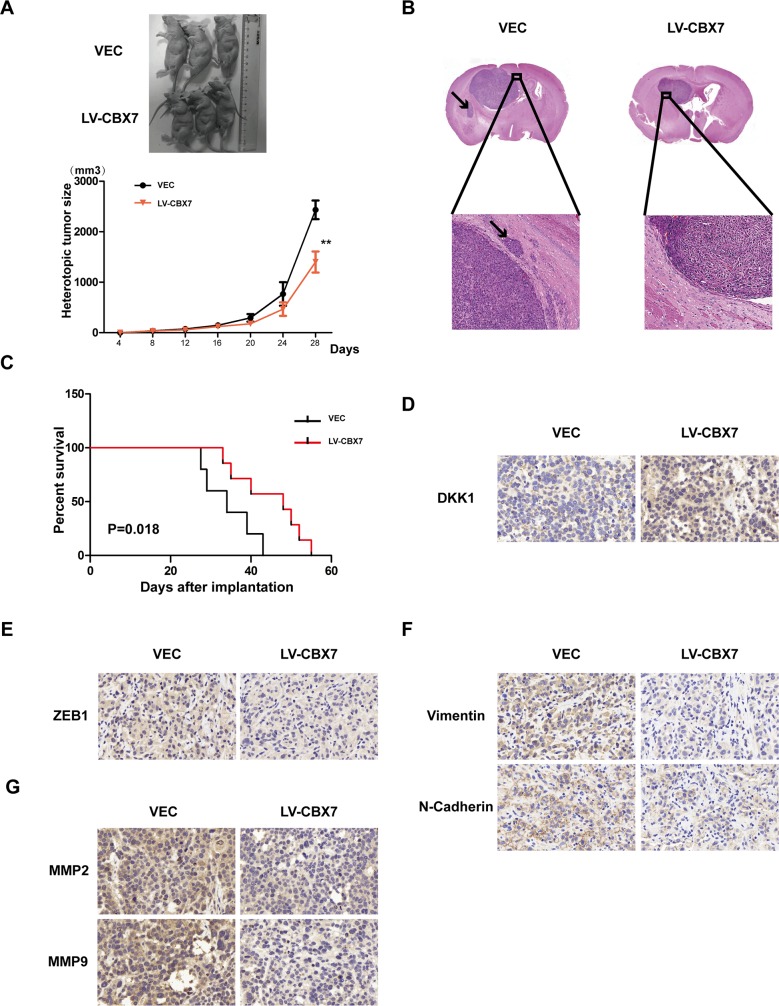
CBX7 function was demonstrated *in vivo* **(A)** LN229/VEC and LN229/CBX7 stabled cells were counted and transplanted subcutaneously in nude mice respectively. After about 4 weeks, xenografted tumors were formed and difference in tumor volume was counted. Size in CBX7 overexpressing groups was smaller than vector groups. **(B)** Cells were counted and transplanted in orthotopic nude mice model. After about 20 days, HE assay was performed and showed that not only the volume became smaller, but the invasion ability became weak in CBX7 overexpressing groups. Arrow sign points to the invasion sites. **(C)** CBX7 enhanced survival ability of nude mice injected in orthotopic. **(D)** CBX7 promote the level of DKK1 *in vivo* tested by immunohistochemical analysis. **(E-G)** EMT-like processes were blocked and the expression of MMPs were reduced *in vivo*, which were analyzed via immunohistochemical analysis.

CBX7 overexpression decreases Wnt/β-catenin signaling and blocks glioma cell invasion mediated by DKK1, which was verified in LN229, T98G and PGC cell lines. Further test should be induced *in vivo*. The vector and CBX7 overexpressing LN229 and PGC were transplanted subcutaneously in nude mice. After 15 days, xenografted tumors were visible, and tumor size was counted and smaller size in CBX7 overexpressing groups (Figure 7A and Supplementary Figure 3A). The same outcome was displayed in orthotopic nude mice model. What's more, cell motion was less in CBX7 overexpressing groups (Figure 7B and Supplementary Figure 3B). So tumor growth including invasion was inhibited after treatment by CBX7. And low CBX7 expression is associated with poor survival (Figure 7C and Supplementary Figure 3C). We also confirmed CBX7-dependent increased DKK1 expression *in vivo* by overexpressing CBX7 in xenograft tumors and performing immunohistochemical analysis of DKK1 (Figure 7D and Supplementary Figure 3D). And the EMT relative marker and MMPs were displayed by immunohistochemical analysis (Figure 7E-7G and Supplementary Figure 3E-3G).

## DISCUSSION

Recently, PcG family members have been found to be active in various cancers and correlated with the survival of tumor cells. Previous studies have demonstrated that CBX7 is involved in multiple cancer types as a tumor suppressor. In several studies on prostatic cancer, CBX family members, including CBX7, were shown to facilitate PRC1-mediated transcriptional repression by targeting the complex to tri-methylated lysine 27 of histone H3, inducing chromatin compaction, DNA methylation and repression of the underlying genes [25]. Furthermore, small hairpin RNA-mediated CBX7 knockdown in breast epithelial and breast cancer cells increased the CD44+/CD242/ESA+ cell population and reinforced self-renewal and tumor-initiating ability [37]. Our study also found lower CBX7 expression in higher grade gliomas. Here, we demonstrated that CBX7 functions as a tumor suppressor in glioma by inhibiting invasion. EMT is related to the acquirement of migration and invasion capabilities through improving mesenchymal phenotypes and motility. These processes also overlap with the acquirement of stem cell properties in differentiated tumor cells. A previous study has indicated that CBX7 overexpression enhanced E-Cadherin activity by interacting with HDAC2 and inhibiting its activity on the E-cadherin promoter in thyroid tumors [29]. Therefore, we speculate that CBX7 can decrease EMT-like processes in glioma through similar mechanisms.

Here, we present data that suggests CBX7 is an important inhibitor of β-catenin activation that blocks tumor cell migration and invasion by blocking EMT. Aberrant Wnt/β-catenin pathway activation has been extensively analyzed in brain tumors and has been shown to contribute to maintaining stem-like cells, resistance to therapies and increasing migration and invasion phenotypes. Unlike other tumor types, no β-catenin mutations have been detected in glioma, excluding these events as drivers of β-catenin stabilization. Based on our data, CBX7 should be added to this growing list of β-catenin regulators in glioma that includes SFRP1, DKK1 and Wif1.

The Wnt/β-catenin pathway is active in glioma, and β-catenin is highly expressed and abundant in the nucleus in LN229, T98G and PGC cells. The β-catenin/TCF/LEF transcriptional complex drives transactivation of Wnt target genes, which promotes EMT. Therefore, β-catenin is a core regulatory factor of migration and invasion in glioma. Nuclear β-catenin was decreased following CBX7 overexpression, suggesting a cytosolic translocation event following restoration of this PcG protein. DKK1 is a known Wnt antagonist that inhibits β-catenin/TCF/LEF complex formation and is expressed at low levels in LN229, T98G and PGC cells. The induction of high DKK1 expression by CBX7 is facilitated by direct CBX7 binding to the DKK1 promoter, as shown by ChIP assays. In this study, we demonstrated that CBX7 increases DKK1 expression, which in turn, inhibits invasion at the transcriptional level by targeting Wnt/β-catenin signaling and decreasing downstream ZEB1 expression both *in vivo* and *in vitro*. Decreased ZEB1 expression is negatively associated with EMT, resulting in the acquirement of cell-cell contacts and decreased motility. Following DKK1 knockdown in CBX7 stable cells, Wnt/β-catenin/ZEB1 activity was restored, and E-Cadherin expression was reduced, while Vimentin, N-Cadherin, MMP2 and MMP9 expression was recovered. These findings suggest a crucial role for CBX7 in glioma tumorigenesis as a novel epigenetic regulator of the Wnt pathway by regulating DKK1 expression. It is noteworthy that CBX7 upregulated E-Cadherin and down-regulated Vimentin, but the effect on N-Cadherin was minimal, especially in T98G cells. N-Cadherin is a marker of a mesenchymal phenotype, and its expression is low when EMT is inhibited. Thus, CBX7 may have slight influences on N-Cadherin expression, which is in accordance with previous studies in thyroid cancer [29]. The same phenomenon was demonstrated for MMP2, an invasion-associated MMP, which was not obviously affected like MMP9 in the T98G cell line.

However, the functions of CBX7 are not uniform; although CBX7 is involved in tumor development as a tumor suppressor in thyroid cancer, breast cancer and gliomas, it has the opposite effect on several other cancer types including lymphomagenesis [38] through enhancing stem cell self-renewal and/or increasing the replicative potential of CSCs. CBX7 may repress transcription of the INK4a/ARF locus and act as an oncogene in these contexts [38]. CBX7 could also be a target for follicular lymphoma in the clinic. What's more, CBX7 in gastric cancer [39] and ovarian cancer [40] were reported as an oncogene, too. Hence, we speculate that the expression and effects of CBX7 are tissue and cell type specific, and future experiments are required to explore the diversity of CBX7 functions among various cancers and the molecular mechanisms involved.

In summary, our results suggest that CBX7 expression is associated with the malignancy grade of glioma and patient outcome. Furthermore, CBX7 targets the Wnt/β-catenin signaling pathway by directly binding DKK1 and indirectly influencing the expression of downstream Wnt target genes, including ZEB1. Through these mechanisms, CBX7 inhibits the invasive abilities of glioma cells as well as other malignant phenotypes. Thus, in consideration of inhibiting neoplastic development, the role of CBX7 in glioma needs to be further investigated.

## MATERIALS AND METHODS

### Human tumor samples

The tumor samples used in the study were obtained from patients who were operated in Jiangsu Province Hospital, the First Affiliated Hospital of Nanjing Medical University. The normal brain tissues (NBT) used as control, comprised of brain tissue obtained during the surgery of arterial aneurysm and intractable epilepsy cases. The use of human gliomas and normal tissues were approved by Research Ethics Committee of Nanjing Medical University (Nanjing, Jiangsu, China) and were performed in accordance with the approved guidelines. The evaluation of the CBX7 level of the tissues was in accordance with the approved guidelines. Informed consents were obtained from the patients. The tissues both tumor and control were snap-frozen in liquid nitrogen and stored at − 80°C.

### Western blot analysis

The proteins were separated on 8%, 10 or 12% SDS-PAGE and then transferred to PVDF membrane (Merck Millipore). Samples belonging to a particular experiment were run in a same gel under same experimental conditions. The membrane was blocked in 5% skim milk for 2 h and then membranes were incubated overnight at 4°C with diluted (1:1000) primary antibodies against CBX7 (ab21873, abcam), N-Cadherin (#13116, CST), E-Cadherin (#1395, CST), Vimentin (#5741, CST), β-catenin (#8480, CST), AKT (#4685, CST), P-AKT (#4060, CST), GSK3β (#12456, CST), P-GSK3β (#5558, CST), ZEB1 (ab124512, abcam), MMP2 (#13132, CST), MMP9 (#13667, CST), DKK1 (#48367,CST), β-actin (AF0003, Beytime), GAPDH (AF0006, Beytime), H3 (#4499, CST) followed by incubation with a horseradish peroxidase-conjugated secondary antibody (1:2000, Santa Cruz Biotechnology, CA, USA) for 2 h. After washing with PBST, membranes were probed using SuperSignal^®^ Maximum Sensitivity Substrate (Thermo Fisher Scientific).

### Cell culture and treatment

Human glioma cells (LN229 and T98G) were obtained from the Chinese Academia Sinica cell repository (Shanghai, China). In March 2016, the tissue taken from a patient with a left tempus glioblastoma was obtained from regions comprising viable tumor cells. Within 2 h after acquisition, the tissue samples were dissociated into single-cell suspensions, washed with Hanks solution (Solarbio, Beijing, China) to remove red blood cells, and the number of cells was counted. The primary cultures were maintained in serum. All primary glioma cells (PGC) were preserved in liquid nitrogen. Cells were maintained in Dulbecco's modified Eagle's medium (DMEM, Gibco) supplemented with 10% fetal bovine serum, and incubated at 37°C with 5% CO2.

### Lentivirus packaging and stable cell lines

Lentivirus carrying CBX7 or vector controls were designed and packaged by Genechem (Shanghai, China). Lentivirus were packaged in HEK-293T cells and collected from the medium supernatant. Stable cell lines were established by infecting lentivirus into LN229, T98G and PGC cells and selected by puromycin.

### Oligonucleotides and transfection conditions

Small interfering RNA (siRNA) oligonucleotides targeting DKK1 were chemically synthesized by GenePharma (Shanghai, China). The sequences of the reagent: 5′ to 3′GAGGAAACCAUCACUGAAATT, 3′ to 5′UUUCAGUGAUGGUUUCCUCTT. Cells were transfected 24h after plating using Lipofectamine 2000 (Invitrogen). Transfection complexes were prepared according to the manufacturer's instructions and added directly to the cells in Opti-MEM reduced serum media (Gibco).

### CCK-8 proliferation assay

The proliferative ability of glioma cells was estimated using CCK-8 (Beyotime, China). The LN229 and T98G cells were seeded into 96-well cell culture plates. Every well contained 4×10^3^ cells in 100 μl culture media. The same treatment to LN229/CBX7 and T98G/CBX7 stabled cell lines. After 24 h, 48 h, 72 h or 96 h, the medium of each well was replaced with 100 μl fresh medium with 10 % CCK8, and then the cells were incubated at 37°C for an additional 2h. The absorbance was measured at 450 nm wavelength.

### Colony suppression assay

Glioma cell lines, LN229/VEC, T98G/VEC, LN229/CBX7 and T98G/CBX7 stabled cells, were counted and plated at a density of 400 cells per well and allowed for colony formation. Cells were incubated at 37°C for 3 weeks with growth media being replaced every third day. Colonies were fixed in chilled methanol for 30 minutes, followed by staining with 0.5% crystal violet for another 30 minutes.

### Wound healing scratch assay

CBX7 stabled and their respective control cells were seeded in 6-well cluster plate (1.2 × 10^6^ cells/well) with 2 ml of complete DMEM. At 24 hours, the monolayers were mechanically disrupted with a sterile toothpick or pipette to produce a clean uniform scratch. The assay was performed in three times. The wells were photographed every 24h to monitor the closing of the wound.

### Transwell assays

CBX7 stabled and their respective control cells at density of 3×10^4^ were transferred to the top of Matrigel-coated invasion chambers (BD Biosciences, San Jose, CA, USA) in serum-free DMEM. DMEM containing 10% FBS was added to the lower chamber. After 24 h of incubation and removal of non-invading cells with rinse, the invading cells were fixed with 4% paraformaldehyde and stained with 0.1% crystal violet. The number of invading cells was manually counted in 5 randomly chosen fields under a microscope and images were captured under ×100 magnification. Experiments were repeated three times.

### Three-dimensional spheroid assays

Established cell lines (LN229 and T98G) and the transfected were cultured to 70% confluence. Cells were seeded at a 0.2 × 10^5^ cells/ml density in 96-well ultra-low adherence plates (#7007, Costar). Over the course of 96 hours these cells were induced to aggregate into a multicellular spheroid with an estimated density of 2,000 cells and then matrigel was added into wells. After 48 h, motion of cells was confirmed as fully formed under light microscopy.

### Immunofluorescence staining

Cells (1 × 10^5^/well) seeded onto a glass bottom cell culture dish(No:801002, NEST Biotechnology Co.LTD) were fixed with cooled methanol for 10 min and incubated in PBS with 0.3% Triton X-100 and 1% bovine serum albumin for 1 h. After blocking, cells were stained with rabbit anti-β-catenin (1:200) at room temperature for 1 h and further reacted with anti-rabbit FITC-conjugated secondary goat antibody (1:300, Jackson) for an additional 1 h. After staining, DAPI (Vector Laboratories, Burlingame, CA, USA) was applied to visualize nuclei and characterized by fluorescence microscopy (Leica DM 5000B; Leica, Wetzlar, Germany).

### Luciferase reporter assay

In order to analyze DKK-1 transcriptional activity, a 1000 base pair (bp) genomic fragment corresponding to the region of the DKK-1 promoter between bases -877 and +123 (pGL3-1kbDKK-1) was generated by PCR and cloned into the pGL3 luciferase reporter vector (Promega, Madison, WI, USA). Cells were cotransfected with the reporter vector and beta-galactosidase cDNA by Lipofectamine 2000. Cells were lysed in lysis buffer provided by a luciferase assay kit (Promega) after 48 h transfection, and assayed for luciferase activity using a MicroLumat Plus LB96V luminometer (Berthold Technologies, Bad Wildbad, Germany). The luciferase activity, expressed as relative light units (RLUs), was normalized to beta-galactosidase activity.

### Chromatin immunoprecipitation assay

Chromatin immunoprecipitation (ChIP) assays were performed following the manufacturer's instructions provided in a ChIP assay kit (Millipore). Briefly, cells were cross-linked with 1% formaldehyde at room temperature for 10 min. The DNA protein complexes were immunoprecipitated with specific antibodies and appropriate protein A/G-agarose (Millipore).

DNA was eluted and purified from the complexes and then amplified by PCR using primers specific for the DKK-1 promoters. The following primer were used for semi-quantitative RT-PCR after ChIP assay: region 5′-CCACTTTGATCTCACGCGTC-3′ and 5′-AGAGAGGGAGGCGAGAGACT-3′.

### Xenograft mouse model

Animal experiments were approved by the Animal Management Rule of the Chinese Ministry of Health (documentation 55, 2001) and were in accordance with the approved guidelines and the experimental protocol of Nanjing Medical University. LN229 cells (1 × 10^6^) expressing CBX7 and vector group genes were subcutaneously injected into 5-week-old male nude mice (Cancer Institute of the Chinese Academy of Medical Science). 15 days after injection, the tumor was visible. And within 4 weeks, the nude mice were sacrificed and the tumor tissues were excised and frozen immediately at −80°C for further study.

### Orthotopic mouse model

Animal experiments were approved by the Animal Management Rule of the Chinese Ministry of Health (documentation 55, 2001) and were in accordance with the approved guidelines and the experimental protocol of Nanjing Medical University (Nanjing, China). All experiments involving mice were provided by The Model Animal Research Center of Nanjing University (Nanjing, China). LN229 cells (2.5× 10^5^) stably expressing CBX7 and vector groups were injected intracranially into the striatum of 5-week-old male nude mice using a stereotactic device (coordinates: 2mm anterior, 2mm lateral, 3mm depth from the dura). 20 days after injection, the nude mice were sacrificed and the tumor tissues were excised and frozen immediately at −80°C for further study.

### Immunohistochemistry

Briefly, fresh specimens were under cryopreservation and routinely processed into frozen sections. Five-micron-thick sections were prepared, and immunohistochemical staining with streptavidin-biotin immunoperoxidase assay was performed using antibodies against DKK1, ZEB1, N-Cadherin, Vimentin, MMP2 and MMP9. Slides were imaged under a light microscope (Leica, German) at 200 or 400 × magnification.

### Statistical analysis

All experiments were performed three times and data were presented as mean ± standard error. Data were analyzed with SPSS 10.0. T-test was used to analyze differences in each two-group comparison, while One-way ANOVA was used to determine the difference among at least three groups. P < 0.05 was considered statistically significant.

## SUPPLEMENTARY FIGURES



## Abbreviations

CNS: Central nervous system
PcG: Polycomb-group
PRC1: Polycomb repressive complex 1
PRC2: Chromobox homolog 7
CBX7: Polycomb repressive complex 2
ESCs: Embryonic stem cells
HSCs: Hematopoietic stem cells
CSC: Cancer stem cell
EMT: Epithelial-to-mesenchymal transition
PGC: the primary glioma cell
ChIP: Chromatin immunoprecipitation
CCK-8: Cell Counting Kit-8
EdU: 5-ethynyl-2´-deoxyuridine
MMP: Matrix metalloproteinase
ECM: Extracellular matrix
siRNA: small interfering RNA
