# Supplementary material for: CBX7 negatively regulates migration and invasion in glioma via Wnt/β-catenin pathway inactivation
Source: Oncotarget. 2017 Mar 28;8(24):39048–63. doi: 10.18632/oncotarget.16587 (PMC5503594; doi:10.18632/oncotarget.16587)
Supplement: Supplementary file 1 [file oncotarget-08-39048-s001.pdf]

CBX7 negatively regulates migration and invasion in glioma via Wnt/β-catenin pathway inactivation

SUPPLEMENTARY FIGURES

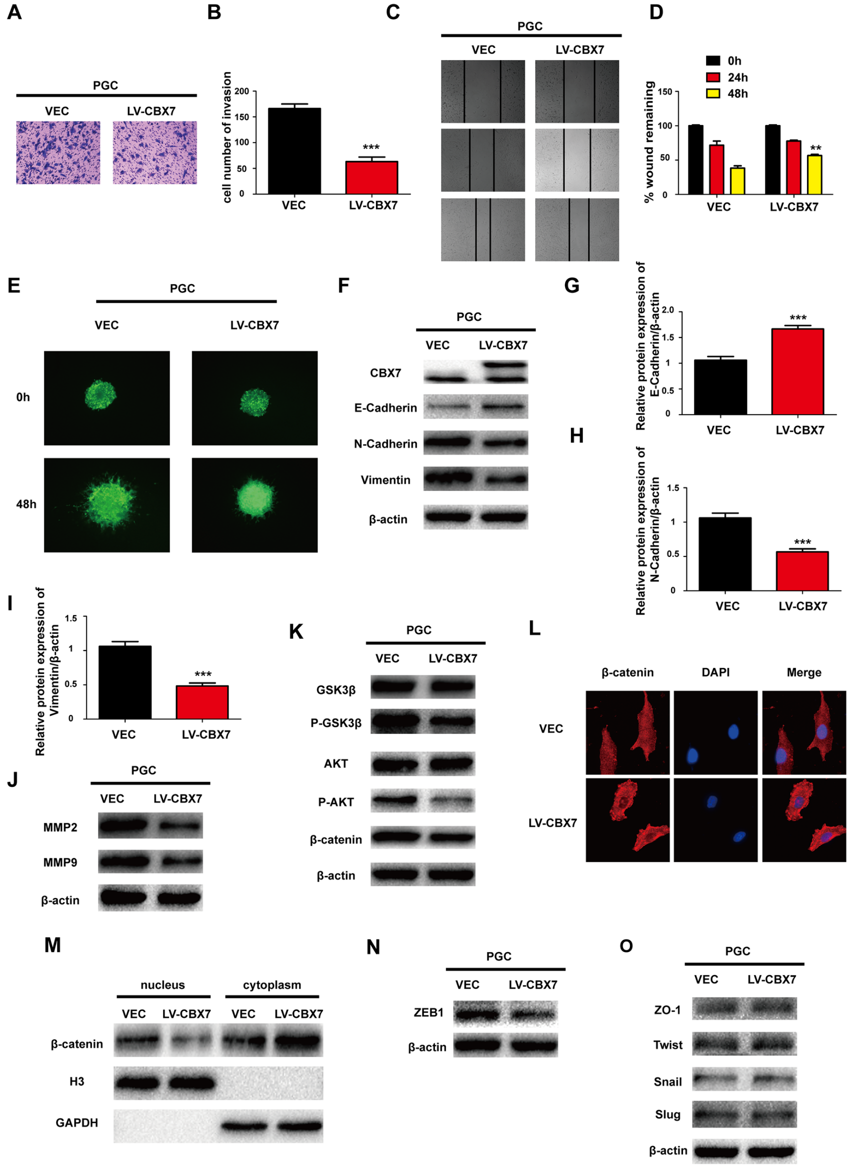

Supplementary Figure 1: CBX7 overexpression inhibits glioma migration and invasion and blocks Wnt/β-catenin pathway in primary glioma cells (PGC). **(A-B)** After transfecting LV-CBX7, debilitated ability of invasion was demonstrated by transwell assay and counted into the bar graph. **(C-D)** After transfecting LV-CBX7, poor ability of migration was detected by wound-healing assay and counted into bar graph. **(E)** Three-dimensional spheroid assays was carried out to display invasive ability after overexpressing CBX7. **(F-I)** Cells were transfected with CBX7, and the levels of cell EMT relative proteins including Vimentin, N-Cadherin, E-Cadherin were detected by western blot analysis. β–actin was used as an endogenous normalizer. **(J)** Western blot assay was used to show decreased expression of MMP2 and MMP9 in PGC/CBX7 stabled cells. β–actin was used as an endogenous normalizer. **(K)** The effect of CBX7 on the Wnt signaling pathway was analyzed by immunoblotting with the indicated antibodies. **(L)** PGC cells were stained with an anti-β-catenin antibody (red) and DAPI (blue). **(M)** Cells were fractionated, and the nuclear and cytoplasmic extracts were subjected to immunoblotting. GAPDH and H3 were used as controls for the cytoplasmic and nuclear, respectively. **(N)** ZEB1 expression in PGC/CBX7 stabled cells were subjected to immunoblotting. β-actin was used as an endogenous normalizer. **(O)** The expression of other EMT markers such as Slug, Snail, Twist and ZO-1 were detected by WB in PGC/CBX7 stabled cells. All experiments were performed in triplicate. * P<0.05; ** P < 0.01 and *** P < 0.001.

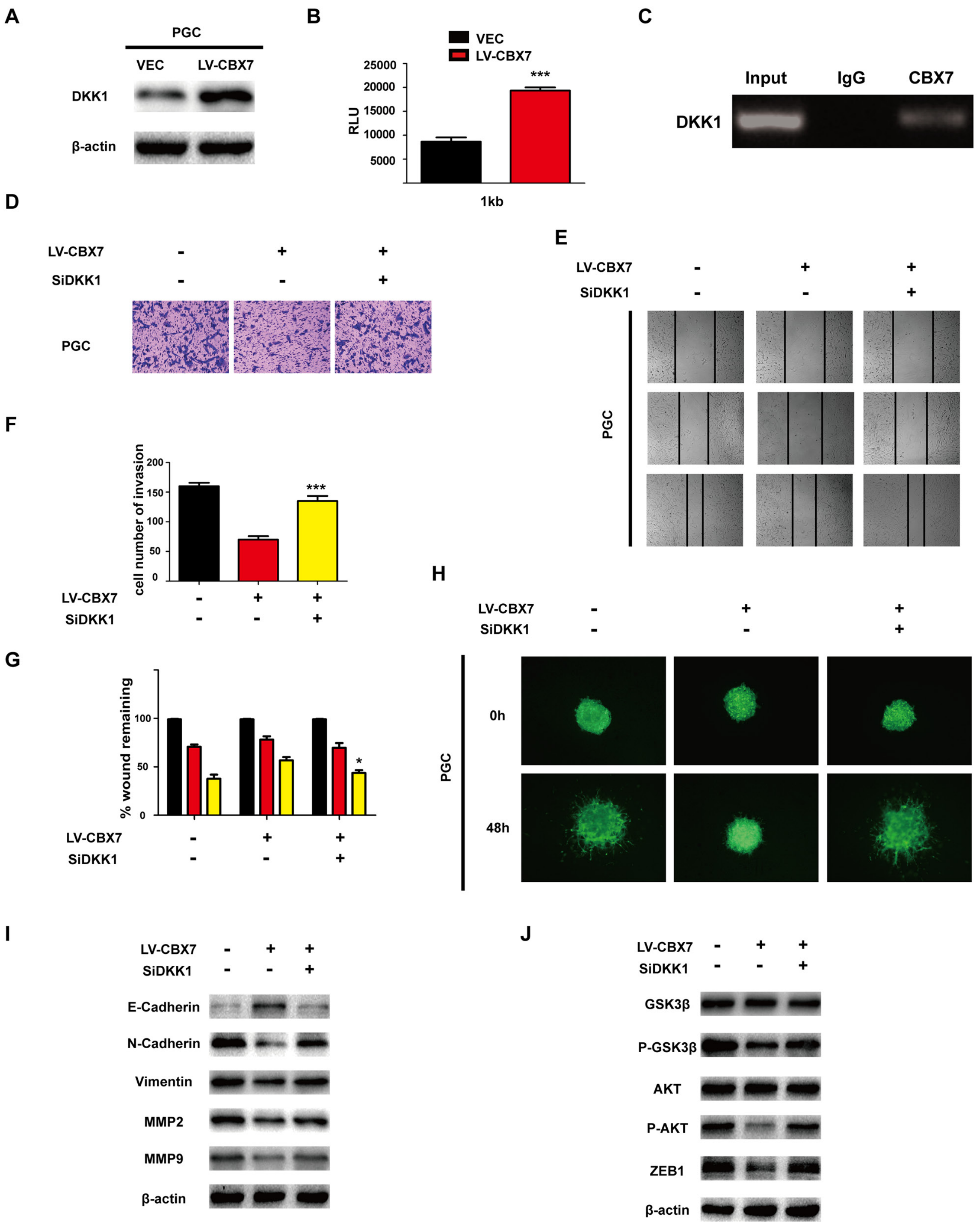

Supplementary Figure 2: DKK1 was key in blocking of Wnt/β-catenin pathway in PGC/CBX7 stabled cells. **(A)** CBX7 impact the expression of DKK1 in PGC, analyzed by immunoblotting with the indicated antibodies. β-actin was used as control. **(B)** A luciferase reporter assay was performed to measure DKK-1 promoter activity in PGC. **(C)**The results of ChIP analysis show the CBX7 was recruited to the DKK-1 promoter regions in PGC cells. IgG was used as an immunoprecipitation control. **(D, F)** A Transwell assay was performed and counted in PGC treated with siDKK1. **(E, G)** A Wound-healing assay was performed and counted in PGC treated with siDKK1. **(H)** A three-dimensional spheroid assay was performed in TGC treated with siDKK1. **(I)** Effect of the inhibition of DKK-1 on CBX7-induced increased activity of the E-Cadherin and oppositively on other invasion related proteins. Cells treated with DKK-1 siRNA were subjected to immunoblotting with appropriate antibodies. **(J)** CBX7-induced reduction of phosphorylated AKT and GSK3-β and ZEB1 were reversed by treatment of siDKK1. β-actin was used as an endogenous normalizer in immunoblotting. All experiments were performed in triplicate. * $P<0.05$; ** $P < 0.01$ and *** $P < 0.001$.

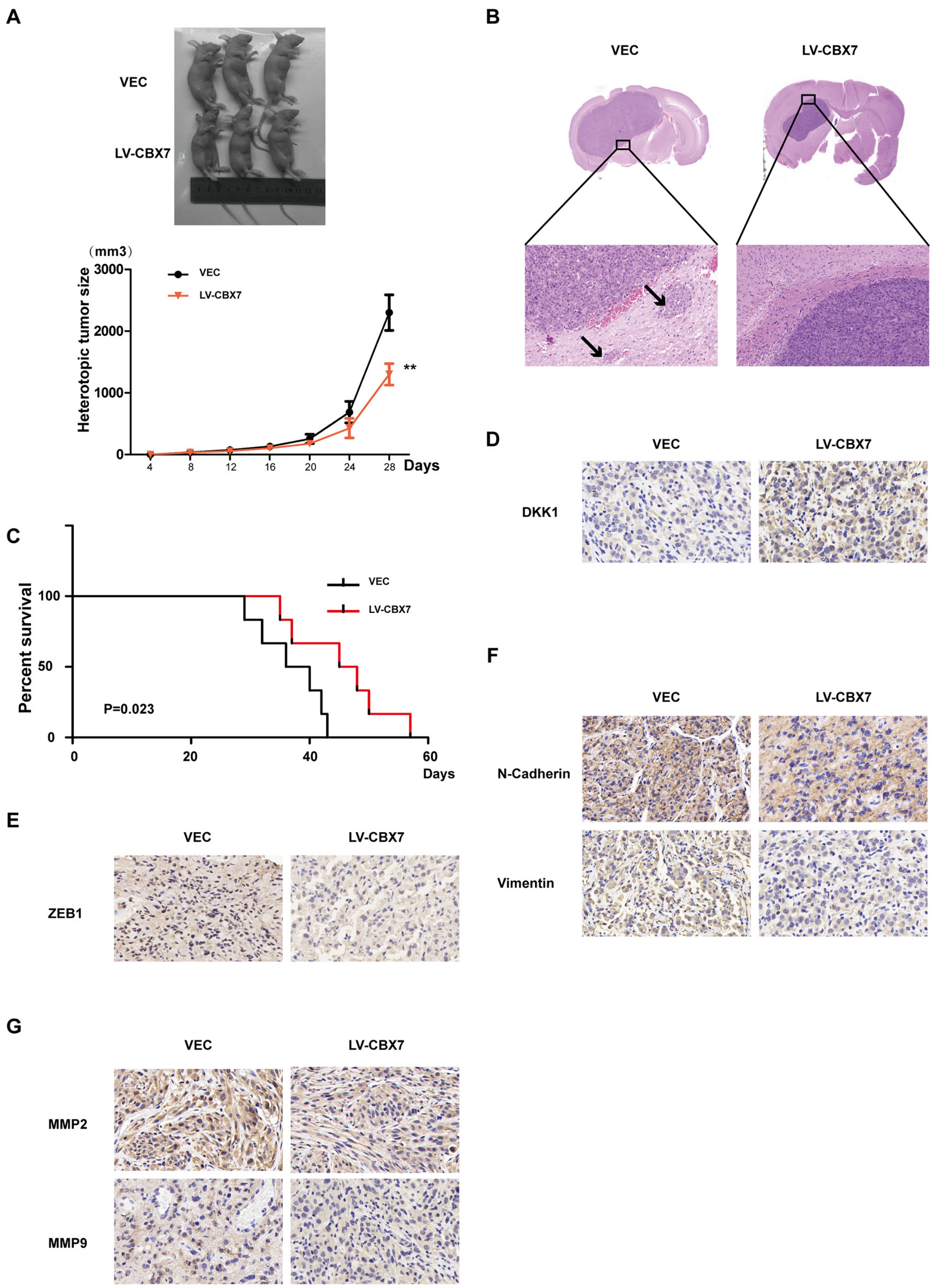

Supplementary Figure 3: CBX7 function was demonstrated *in vivo* using PGC. **(A)** PGC/VEC and PGC/CBX7 stabled cells were counted and transplanted subcutaneously in nude mice respectively. After about 4 weeks, xenografted tumors were formed and difference in tumor volume was counted. Size in CBX7 overexpressing groups was smaller than vector groups. **(B)** Cells were counted and transplanted in orthotopic nude mice model. After about 20 days, HE assay was performed and showed that not only the volume became smaller, but the invasion ability became weak in CBX7 overexpressing groups. Arrow sign points to the invasion sites. **(C)** CBX7 enhanced survival ability of nude mice injected in orthotopic. **(D)** CBX7 promoted the level of DKK1 *in vivo* tested by immunohistochemical analysis. **(E-G)** EMT-like processes were blocked and the expression of MMPs was reduced *in vivo*, which were analyzed via immunohistochemical analysis. All experiments were performed in triplicate. * $P<0.05$; ** $P < 0.01$ and *** $P < 0.001$.

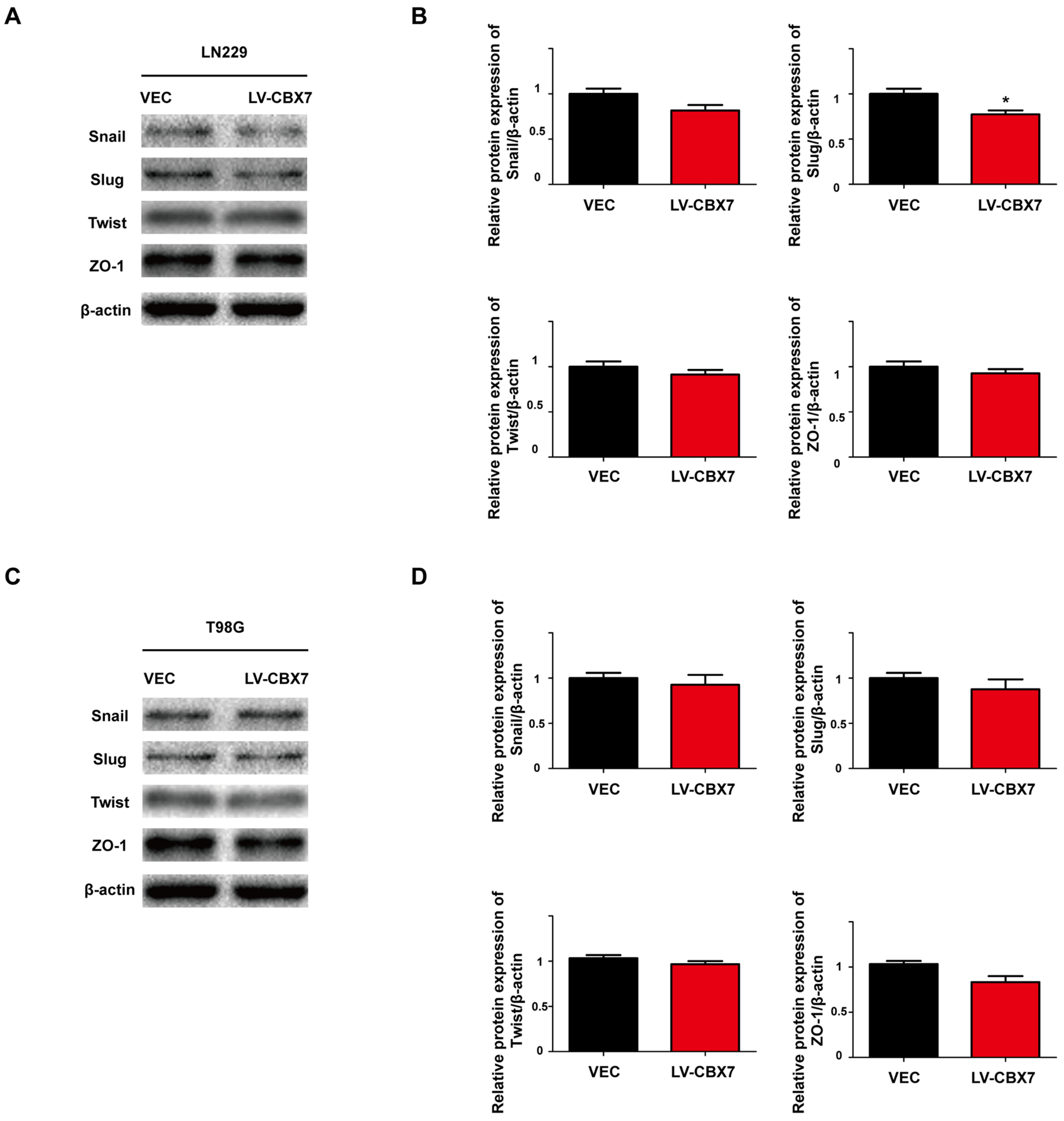

Supplementary Figure 4: The other EMT markers except ZEB1 were detected via immunoblotting with appropriate antibodies in LN229 and T98G cell lines. (A-B) After overexpression of CBX7, protein expression of Slug, Snail, Twist and ZO-1 in LN229 was analyzed by immunoblotting and counted. β-actin was used as control. **(C-D)** After overexpression of CBX7, protein expression of Slug, Snail, Twist and ZO-1 in T98G was analyzed by immunoblotting and counted. β-actin was used as control. All experiments were performed in triplicate. * $P<0.05$; ** $P < 0.01$ and *** $P < 0.001$.
